# Identification of a new HIV-1 circulating recombinant form (CRF159_01103) derived from CRF103_01B and CRF01_AE in Hebei Province, China

**DOI:** 10.1038/s41598-024-64156-8

**Published:** 2024-06-08

**Authors:** Weiguang Fan, Zhen Zhang, Haoxi Shi, Jianru Jia, Penghui Shi, Sisi Chen, Xinli Lu

**Affiliations:** 1 Clinical Laboratory of the People’s Hospital of Baoding, Baoding, 071000 Hebei China; 2 Infection Division of the People’s Hospital of Baoding, Baoding, 071000 Hebei China; 3 Department of AIDS Research, Hebei Provincial Center for Disease Control and Prevention, Shijiazhuang, 050021 Hebei China

**Keywords:** HIV-1, Circulating recombinant forms, CRF159_01103, Hebei, Genetics, Immunology, Molecular biology

## Abstract

Recombinant HIV-1 genomes identified in three or more epidemiological unrelated individuals are defined as circulating recombinant forms (CRFs). CRFs can further recombine with other pure subtypes or recombinants to produce secondary recombinants. In this study, a new HIV-1 intersubtype CRF, designated CRF159_01103, isolated from three men who have sex with men with no epidemiological linkage, was identified in Baoding city, Hebei Province, China. CRF159_01103 was derived from CRF103_01B and CRF01_AE. Bayesian molecular clock analysis was performed on the CRF01-AE and CRF103_01B regions of CRF159_01103. The time of origin of CRF159_01103 was predicted to be 2018–2019, indicating that it is a recent recombinant virus. The emergence of CRF159_01103 has increased the complexity of the HIV-1 epidemic in Hebei Province.

## Introduction

As a result of the high mutation, recombination and replication rates of HIV-1, its recombinant forms are becoming increasingly complex.^1^ The coinfection and recombination of different HIV-1 subtypes create many opportunities for the emergence of new recombinant forms. To date, more than 150 circulating recombinant forms (CRFs) and numerous unique recombinant forms (URFs) have been identified by the analysis of near-full-length genomes (NFLGs) globally. Furthermore, a new CRF will emerge if more than three URFs with the same recombinant structure are found in different areas with no epidemiological linkage. Compared with other countries in the world, China has a high proportion of HIV-1 recombinant strains^1^.

In Hebei, CRF01_AE, CRF07_BC and subtype B were the dominant HIV-1 subtypes, similar to the dominant subtypes across the whole of China^2,3^ .However, recently, many other subtypes such as CRFs_0107, URFs_0107, CRFs_01B and URFs_01B have been continually identified in Hebei among the sexually-active population, especially among men who have sex with men (MSM). For example, CRF80_0107, CRF103_01B and CRF123_0107 were detected circulating in the MSM population in 2019, 2020 and 2022, respectively^4–6^_._

Baoding is the second largest city with HIV/AIDS cases in Hebei Province, and MSM are the most affected population^7^. Over the past three years, two new CRFs (CRF103_01B and CRF123_0107) and many URFs have been reported in Baoding^5,6^_._ However, no novel second-generation recombinant CRFs were confirmed among the minor subtypes. In this study, we identified a new HIV-1s-generation circulating recombinant CRF159_01103 composed of CRF103_01B and CRF01_AE among MSM in Baoding, Hebei Province, and analyzed its evolutionary history.

## Results

**Figure 1:**
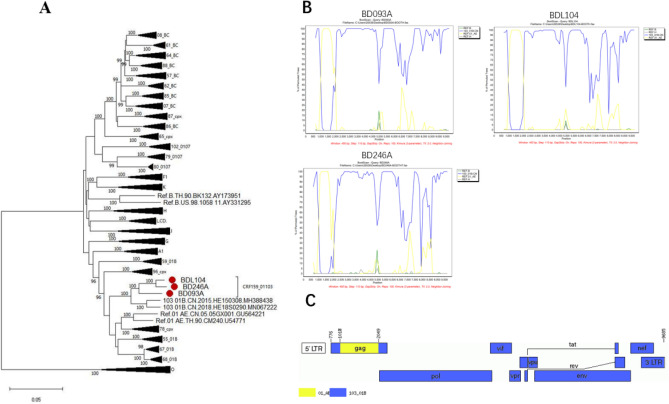
Phylogenetic and recombination analyses based on the near-full-length genome sequences of CRF159_01103. (**A**) Representative HIV-1 CRF reference sequences were used to construct a Maximum-likelihood (ML) phylogenetic tree. The sequence of CRF159_01103 (BD093A, BDL104, BD246A) is marked with a red filled circle. The NFLG phylogenetic tree was constructed by the FastTree V2.1.10 using the approximately maximum likelihood method with the general time reversible (GTR) model and adjusted with Figtree V1.4.4. Bootstrap values greater than 0.9 were considered stable. The scale bar represents 5% genetic distance. (**B**) Bootscanning analysis of CRF159_01103. Bootscanning analysis was conducted using a window size of 400 bp and a step size of 100 bp along with reference sequences of CRF01_AE (yellow line), CRF103_01B (blue line), subtype B (green line) and subtype H (black line). (**C**) Genetic map of CRF159_01103. A mosaic map was generated using the Recombinant HIV-1 Drawing Tool (https://www.hiv.lanl.gov/components/sequence/HIV/crfdb/crfs.comp). The CRF01_AE segment is displayed in yellow, and the CRF103_01B segments are displayed in blue.

**Figure 2:**
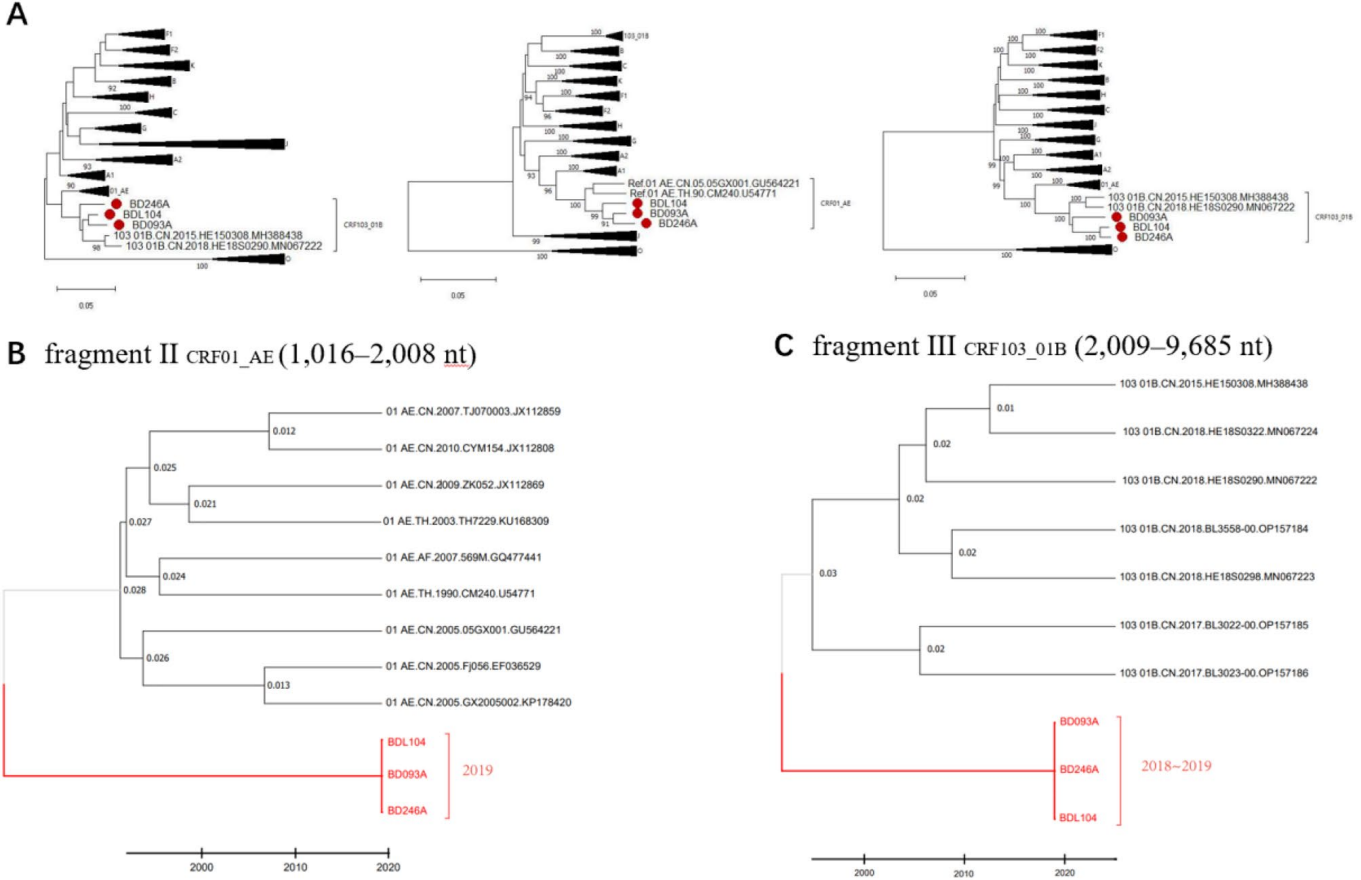
Phylogenetic and evolutionary analysis of the subregions from CRF159_01103. (**A**) Utilizing the FastTree2.1.10 software, maximum likelihood phylogenetic trees were constructed for each subtype sequence using the maximum likelihood method (ML). The GTR + CAT, SPR = 4 model was selected as the alternative model. The Shimodaira-Hasegawa method was employed for branch support. (**B**) and (**C**) MCC trees of the longest II_CRF01_AE_ region (1016–2008 in HXB2) and III _CRF103_01B_ region (2009–9685 in HXB2). TempEst was employed to ascertain the sufficiency of temporal signals in the subsets for the estimation of molecular clock phylogenies. Time phylogenies for the CRF01_AE and CRF103_01B subsets were constructed in BEAST 1.8.4, utilizing an uncorrelated log-normal relaxed clock, a Bayesian skygrid coalescent model, and a GTR + G4 substitution model. Sequences of CRF159_01103 were marked as red branches.

The NFLG length sizes for BD093A, BD246A and BDL104 were 8498, 8896 and 8879 nt, respectively, spanning from the *gag* gene to the partial gene region of the 3ʹ-long terminal repeat (LTR) (position based on HXB2 numbering: 665–9658). Maximum-likelihood (ML) phylogenetic tree based on NFLGs showed that BD093A, BD246A and BDL104 formed an unusual monophyletic cluster with a bootstrap value of 100% (Fig. 1a), separate from other reference subtypes, indicating a potential novel CRF. We further analyzed the gene maps by bootscanning (Fig. 1b) and jpHMM (Fig. 1c) and found that these three NFLGs had the same recombination patterns comprising CRF103_01B and CRF01_AE. They contained one CRF01_AE fragment inserted into the CRF103_01B backbone (Fig. 1c). This backbone was divided into three subregions, including two CRF103_01B subregions and one CRF01_AE region: I CRF103_01B (HXB2, 790–1015 nt), II CRF01_AE (HXB2, 1016–2008 nt), and III CRF103_01B (HXB2, 2009–9685 nt). Their parental linkages were confirmed using subregion phylogenetic analysis (Fig. 2a). In summary, according to the standardized naming recommendations for HIV-1, the novel recombinant lineage was designated CRF159_01103. The gene sequences of BD093A, BD246A and BDL104 were deposited in the GenBank database under accession numbers PP056116, PP056117 and PP056118, respectively.

To determine the time of origin of CRF159_01103, Bayesian molecular clock analysis was performed using the CRF01_AE region (region II) and CRF103_01B region (region III) of CRF159_01103 to estimate the duration of time of the most recent common ancestor (tMRCA). TempEst was employed to ascertain the sufficiency of temporal signals in the subsets for the estimation of molecular clock phylogenies. Time phylogenies for the CRF01_AE and CRF103_01B subsets were constructed in BEAST 1.8.4, utilizing an uncorrelated log-normal relaxed clock, a Bayesian skygrid coalescent model, and a GTR + G4 substitution model. As shown in Fig. 2b and c, the tMRCA of the CRF01_ AE and CRF103_01B gene segments within CRF159_01103 emerged in the years 2019 and 2018–2019, respectively. This confirmed that CRF159_01103 is a recent recombinant virus.

## Discussion

In the 1990s, CRF01_AE was introduced into China and was detected among the sexually-active population^8^. Following its spread, it became the predominant epidemic subtype together with CRF07_BC among MSM in China^9^. CRF01_AE is the most frequent constituent of URFs found in Hebei, China^10^. One study reported that CRF103_01B originated from the MSM population in Beijing around March 2002 to April 2006 and continued to spread at a low level among the MSM population, then being transmitted to the wider population in northern China through heterosexual contact^11^. The CRF103_01B strain was first identified through NFLG analysis among MSM in the cities of Baoding and Shijiazhuang, Hebei Province, China, in 2020. In 2016, Lu^12^ showed that four CRF01_AE/B recombinant strains contained the same breakpoints as CRF103_01B in the *gag* gene and partial *pol* gene region, which suggested that CRF103_01B had been circulating among MSM before the identification of CRF103_01B. In this study, the dual infection of CRF103_01B and CRF01_AE among MSM resulted in the emergence of the second-generation recombinant CRF159_01103, suggesting that CRF103_01B rapidly spread. To our knowledge, CRF159_01103 is the third new HIV-1 CRF discovered in Hebei Province, China. Multiple studies worldwide have shown that HIV-1 recombinant strains have significantly enhanced viral adaptability and are more prone to produce drug-resistant site mutations^13–17^. In addition, due to the differences in HIV-1 recombinant forms between different regions, understanding HIV-1 variants and diversity at the region level can effectively formulate and develop vaccines, and may bring the greatest benefits to specific countries and regions^18,19^. Therefore, continuous monitoring the occurrence and spread of HIV-1 recombinant strains is of great practical significance for the prevention and control of HIV/AIDS.

In conclusion, we identified a new HIV-1s-generation CRF159_01103 composed of CRF103_01B and CRF01_AE. It is estimated that CRF159_01103 originated in the years 2018–2019. The emergence of CRF159_01103 indicates that the local HIV-1 epidemic is complex, especially among MSM.

## Materials and methods

We obtained plasma samples from three HIV-1-infected MSM (BD093A, BD246A and BDL104) in Baoding city, Hebei Province, China. Epidemiological linkage was not observed according to the baseline epidemiological data for these three individuals and their basic epidemiological information are showed in Table 1.

**Table 1:** The epidemiological information of the three individuals.

Strain name	Sampling year	Sampling region	Gender	Age (years)	Transmission route	CD4 + value (cells/mL)
BD093A	2019	Baoding, Hebei	Male	32	MSM	339
BD246A	2023	Baoding, Hebei	Male	27	MSM	32
BDL104	2022	Baoding, Hebei	Male	52	MSM	60

HIV-1 viral RNA extraction, NFLG amplification, sequencing and sequence assembly were completed according to previously published methods^20^. Standard reference sequences for HIV-1 subtypes were downloaded from the HIV database (www.hiv.lanl.gov/content/index). Multiple sequence comparisons and manual editing with ClustalW were then performed using Bio-Edit 7.0 software. The NFLG phylogenetic tree was constructed by the FastTree V2.1.10 based on the approximately maximum likelihood method with the general time reversible (GTR) model and adjusted with Figtree V1.4.4.Then, online jpHMM and SimPlot 3.5.1 software were used for similarity mapping and Bootscan analysis to identify the recombination breakpoints. Finally, to better understand the evolutionary history of CRF159_01103, the Bayesian phylogenetic approach was performed in BEAST 1. 8.4.

### Statement

This experimental protocol was approved by the Ethics Committee of Baoding People’s Hospital (protocol number: 2019-03), and all the methods in this study were performed in accordance with the approved guidelines.

### Ethical issues

All analyses were performed on de-identified datasets to protect participants’ anonymity. Informed consent was waived by Baoding People’s Hospital Ethics committee because no participant`s privacy such as name was involved in this study.

### Supplementary Information

Supplementary Information.

## Data Availability

The gene sequences of BD093A, BD246A and BDL104 were deposited in the GenBank database under accession numbers PP056116, PP056117 and PP056118, respectively.
