# Supplementary material for: Identification of a new HIV-1 circulating recombinant form (CRF159_01103) derived from CRF103_01B and CRF01_AE in Hebei Province, China
Source: Sci Rep. 2024 Jun 8;14:13182. doi: 10.1038/s41598-024-64156-8 (PMC11161575; doi:10.1038/s41598-024-64156-8)

**Datasets for Phylogenetic Analysis in Figs. 1 and 2.**

In phylogenetic analysis, using the Cluster W program in Bioedit software to compare the NFLG sequence with various reference subtypes of HIV-1 M group strains (more than 50 strains such as CRF01_AE, CRF103_01B, CRF96_cpx) and HIV-1 O group reference sequences.


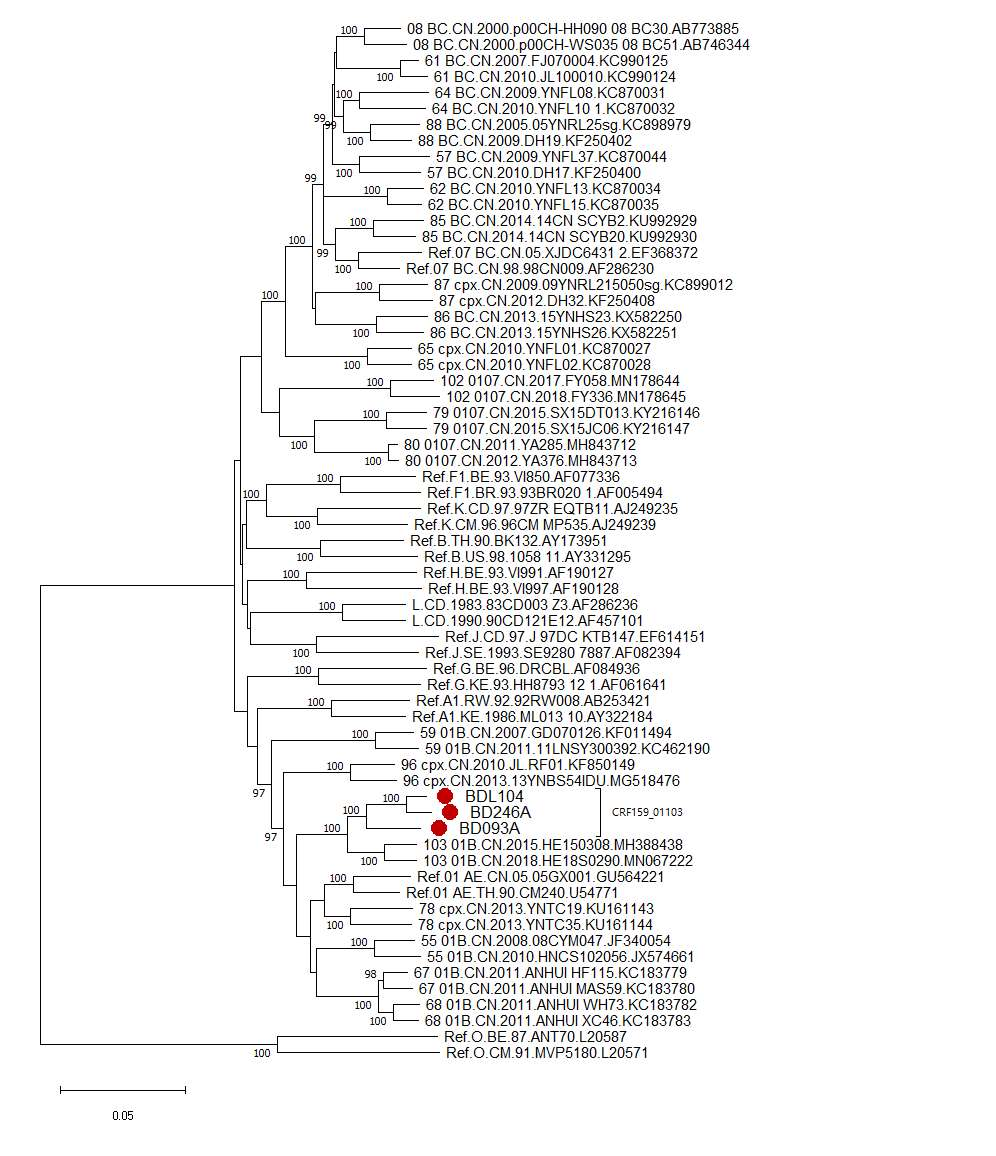


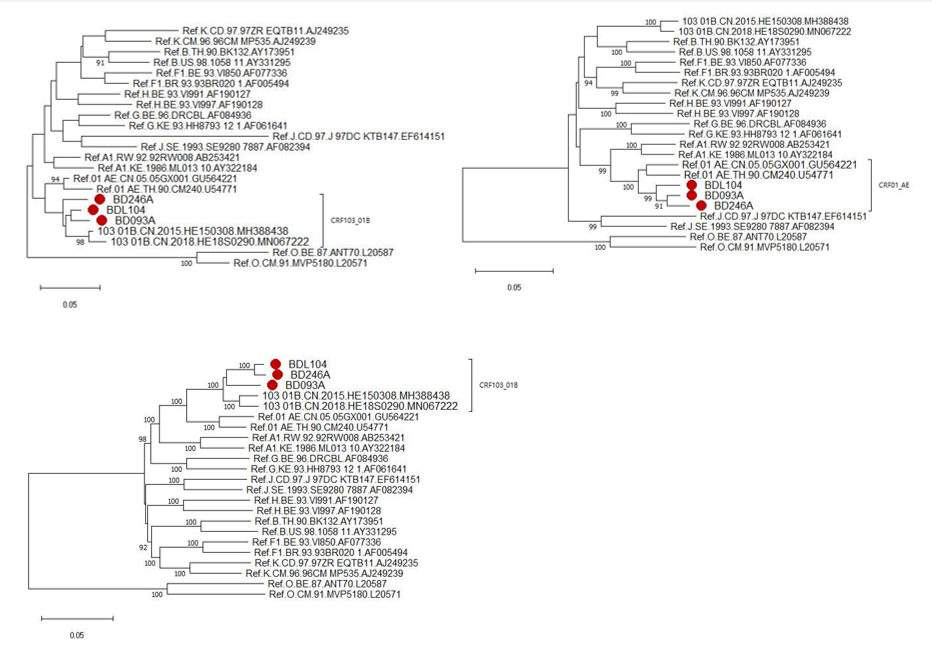


**Datasets for Bayesian phylogenetics performed with BEAST**

TempEst was employed to ascertain the sufficiency of temporal signals in the subsets for the estimation of molecular clock phylogenies. Time phylogenies for the CRF 01_AE and CRF103_01B subsets were constructed in BEAST 1.8.4, utilizing an uncorrelated log-normal relaxed clock, a Bayesian skygrid coalescent model, and a GTR+G4 substitution model. The reference sequence used 9 CRF01_AE sequences including CN2005.GX2005002.KP178420, as well as 7 CRF103_01B sequences including CN.2017.BL3023-00.OP157188.


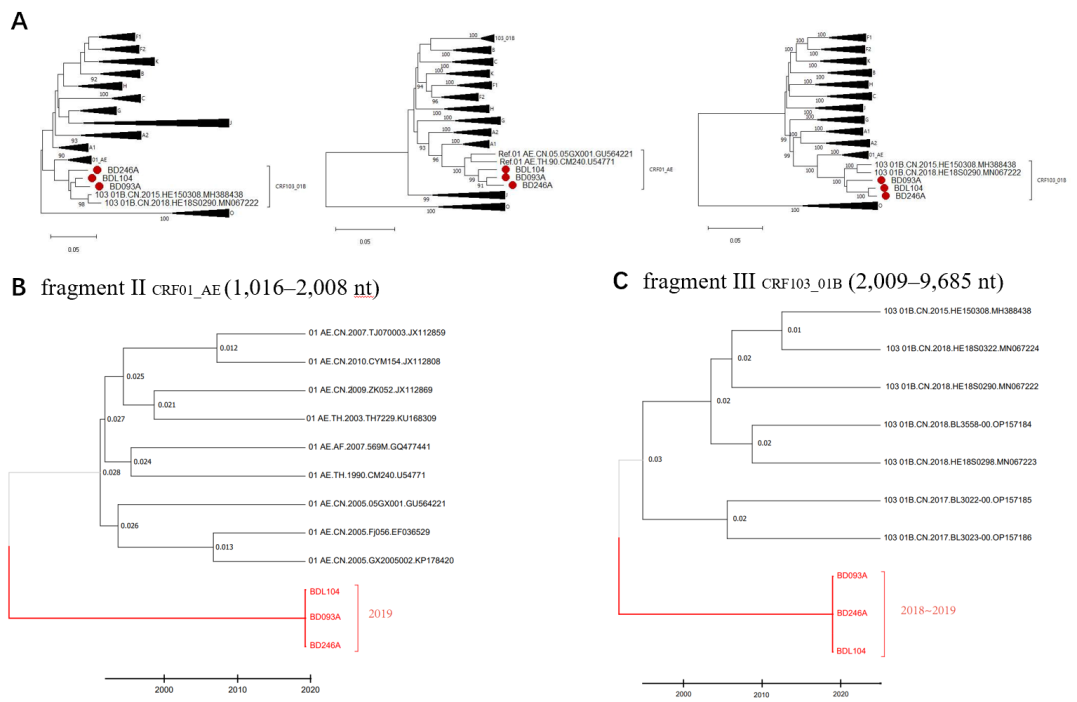

Supplement: Supplementary file 1 — Supplementary Information. [file 41598_2024_64156_MOESM1_ESM.docx]
